# OmicsAnalyst: a comprehensive web-based platform for visual analytics of multi-omics data

**DOI:** 10.1093/nar/gkab394

**Published:** 2021-05-21

**Authors:** Guangyan Zhou, Jessica Ewald, Jianguo Xia

**Affiliations:** Institute of Parasitology, McGill University, Montreal, Quebec, Canada; Department of Natural Resource Sciences, McGill University, Montreal, Quebec, Canada; Institute of Parasitology, McGill University, Montreal, Quebec, Canada; Department of Animal Science, McGill University, Montreal, Quebec, Canada

## Abstract

Data analysis and interpretation remain a critical bottleneck in current multi-omics studies. Here, we introduce OmicsAnalyst, a user-friendly, web-based platform that allows users to perform a wide range of well-established data-driven approaches for multi-omics integration, and visually explore their results in a clear and meaningful manner. To help navigate complex landscapes of multi-omics analysis, these approaches are organized into three visual analytics tracks: (i) the *correlation network analysis* track, where users choose among univariate and multivariate methods to identify important features and explore their relationships in 2D or 3D networks; (ii) the *cluster heatmap analysis* track, where users apply several cutting-edge multi-view clustering algorithms and explore their results via interactive heatmaps; and (iii) the *dimension reduction analysis* track, where users choose among several recent multivariate techniques to reveal global data structures, and explore corresponding scores, loadings and biplots in interactive 3D scatter plots. The three visual analytics tracks are equipped with comprehensive options for parameter customization, view customization and targeted analysis. OmicsAnalyst lowers the access barriers to many well-established methods for multi-omics integration via novel visual analytics. It is freely available at https://www.omicsanalyst.ca.

## Graphical Abstract

**Graphical Abstract:**
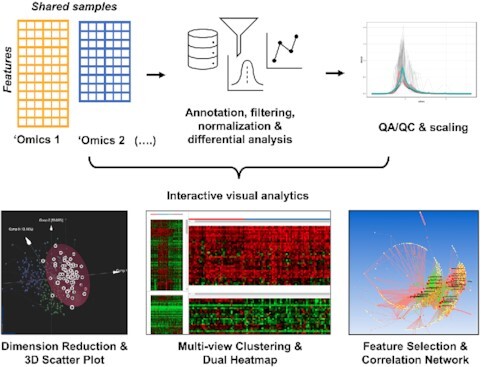
Multi-omics integration using OmicsAnalyst. Individual omics datasets need to be uploaded and processed first. After quality check and proper normalization, different multi-omics integration strategies can be applied. The results can be visually explored in the form of networks, heatmaps and scatter plots.

## INTRODUCTION

The rapid development and increasing accessibility of various omics profiling technologies such as massive parallel sequencing and mass spectrometry have made multi-omics data collection more routine practices in recent years. These multi-omics studies promise to provide more holistic pictures to enable comprehensive understanding of complex diseases and biological processes (1,2). As a result, the last few years have witnessed a growing number of bioinformatics tools and statistical methods developed for multi-omics integration (3,4). These computational approaches can be largely classified as either knowledge-driven or data-driven strategies. The knowledge-driven strategy is well established. A typical example is to map genes and metabolites of interest into known metabolic pathways or networks and then visually explore the results for hypothesis generation (5–7). A key limitation of this strategy is its dependency on a prior knowledge base. Data analysis and interpretation will be conducted within the confines of this knowledge domain, making it unsuitable for novel discoveries and applications to non-model organisms. The data-driven strategy, on the other hand, depends primarily on the datasets themselves, and can be applied in a more general and unbiased manner (8).

Many different data-driven approaches have been proposed and practiced for multi-omics integration. They can be loosely put into three categories based on their main themes, including (i) *Feature correlationanalysis -*this theme aims to identify features that are correlated across different omics layers and/or co-vary under the conditions of interest. These correlated features provide more detailed delineations of underlying biological processes than those obtained from a single omics layer; (ii) *Sample clusteringanalysis -*this theme aims to leverage multiple molecular profiles to improve sample characterization, such as to identify subsets of cancer patients for more targeted treatments (9); (iii) *Understandingglobal structure -*this theme aims to gain a high-level overview of multi-omics data by extracting and examining their shared structural variations and local patterns. Compared to the knowledge-driven strategy where many user-friendly tools are available, most data-driven methods are in the form of complex multivariate statistics or machine learning algorithms, available mainly in the form of command line programs (10–14). For most researchers, they are harder to use and the results are harder to interpret. User-friendly bioinformatics tools supporting data-driven strategy are urgently needed to help convert the complex multi-omics data into meaningful patterns and insights.

Here, we introduce OmicsAnalyst, a web-based visual analytics platform dedicated for data-driven multi-omics integration. It currently supports more than a dozen well-established methods through three visual analytics tracks - correlation network analysis, cluster heatmap analysis, and dimension reduction analysis. These three visualization tracks are equipped with comprehensive functions and menus to allow users to perform parameter customization, visual exploration and interactive analysis. To help users navigate the tool, we have compiled a comprehensive list of frequently asked questions (FAQs), four different screenshot tutorials, and a case study. The main features of OmicsAnalyst are described below.

### Overview of omicsanalyst

**Figure 1.**
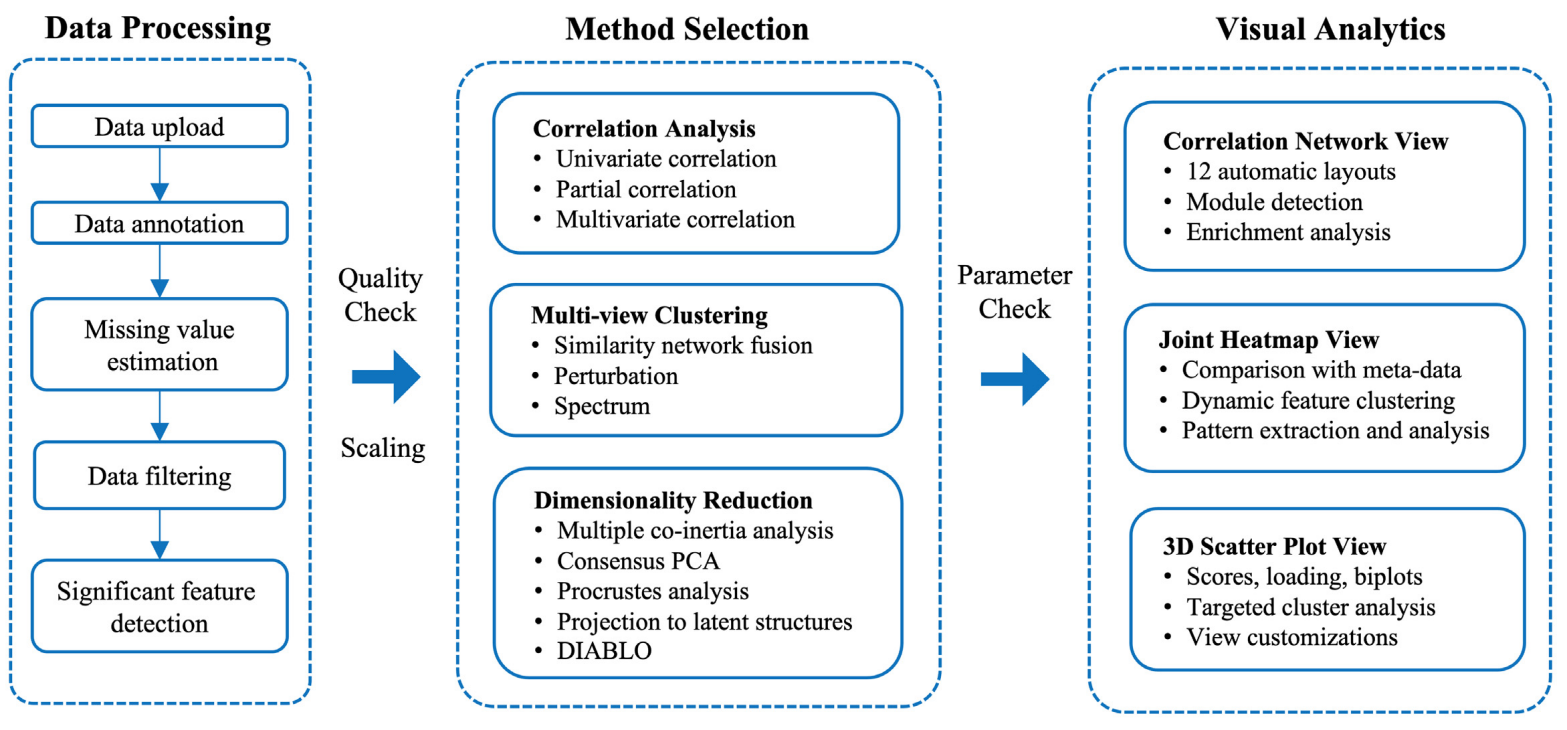
Overall workflow of OmicsAnalyst. Multi-omics integration is divided into three main phases - data processing, method selection and visual analytics. Each phase contains multiple steps and options to allow comprehensive analysis and customization.

The workflow of OmicsAnalyst is shown in Figure 1. It consists of three main phases to help users to navigate the complex procedures of multi-omics analysis. In the Phase 1 (data processing), users go through the conventional single omics data analysis workflow including data upload, annotation, missing value estimation, data filtering, and identification of significant features. After basic quality check and optional data normalization for multi-omics integration, users enter the Phase 2 (method selection). OmicsAnalyst offers a wide array of approaches organized under three categories: correlation network analysis, cluster heatmap analysis, and dimension reduction analysis. After method selection, users are presented with an overview and diagnostic plots to decide whether the default parameters (if any) should be updated. Finally, users enter the Phase 3 (visual analytics) and explore the results through interactive visualization coupled with various statistical and functional analysis.

### Data processing

#### Data upload and annotation

OmicsAnalyst accepts data tables containing feature abundance values (raw or normalized) generated from different omics platforms. They must share the same sample names and metadata information. For data from human and mouse, users can further perform feature annotation for transcriptomics, proteomics, metabolomics and miRNA. The annotation is required for enrichment analysis in the visual analytics stage. *Missing value estimation*. Omics data often contain missing values which could cause potential issues in downstream analysis. Users can exclude features with too many missing values or perform missing value estimation based on several widely used methods. *Data filtering*. Given the high-dimensional nature of omics data, it is strongly recommended to perform unspecific data filtering to exclude features that are unlikely to be useful in downstream analysis. In particular, features that are relatively consistent can be safely excluded based on their inter-quantile ranges (IQRs) or other variance measures. Features that are of very low abundance should also be excluded, as they contribute little to the overall variance-covariance structure in multi-omics integration. *Differential analysis*. Users can perform conventional statistical comparisons to identify significant features within individual omics data. These features will be available for correlation network creation or highlighted in heatmaps or scatter plots. *Quality checking and normalization/scaling*. The goal is to make different omics data more ‘integrable’ by sharing similar distributions. Users can visually examine the distribution of individual omics data through density plot, principal component analysis (PCA) plot, and t-distributed stochastic neighbor embedding (t-SNE) plot. Based on the visual assessment, users can choose among a variety of data transformation, centering and scaling options to improve the integrability.

### Correlation network analysis track

The objective of the correlation network analysis is to identify and visualize relationships between key features from two omics datasets. It consists of three main steps, detailed below.

#### Network creation

This step involves selecting the key features and computing their pairwise correlations. By default, significant features identified by differential analysis during the data processing phase will be used for network creation. However, users can also select top features based on the loading scores from the multivariate dimension reduction methods. Details on the dimension reduction techniques can be found in the ‘Dimension Reduction Analysis Track’ section. The next step is to compute pairwise similarities between selected features. Due to their simplicity and widespread familiarity, univariate methods, such as Pearson correlation, are usually computed as a first line of analysis. However, these methods can produce many false connections due to presence of highly collinear features in omics data. Partial correlation, a multivariate method that measures the correlation between two variables while controlling for all others, has been successfully applied to omics data to detect connections between features that are more likely to represent true dependencies (15).

#### Network customization

Networks with a large number of nodes and edges are too complex and overwhelming for visualization and interpretation. OmicsAnalyst partially addresses this issue by allowing users to control network sizes based on the strengths of correlations. However, applying a single threshold can often produce networks with the majority of edges existing between nodes of the same omics type. This is because in many cases, correlations between features of the same omics type are categorically higher than those of different omics types, likely due to technical differences between platforms. To address this issue, OmicsAnalyst offers two filters to control correlation strengths, one for within-omics and the other for between-omics, with a more stringent default threshold for the former. In addition, users can also apply degree or betweenness filters to control network size based purely on the topological properties of the nodes.

#### Network visual analytics

In addition to providing different filters to allow users to refine the nodes and edges that comprise the network, OmicsAnalyst offers a variety of simple and advanced functions to facilitate visual identification of important network structures. For instance, binary edge coloring is used to differentiate positive and negative correlations, and edge thickness is used to reflect strengths of the correlation to enable quick identification of feature pairs that are highly correlated. OmicsAnalyst also offers 3D network visualization for a deeper perspective of the relationships. Advanced graph layout algorithms, for example edge bundling can be applied to aggregate similar edges into groups to reduce clutter in visualization. Other features such as the concentric circular layout facilitate the evaluation of focal nodes and hierarchical relationships within network. When features are annotated during data processing, users can perform enrichment analysis on a group of nodes selected either manually or through automatic module detection algorithms.

### Cluster heatmap analysis track

The objective of the track is to identify and visually explore relationships between samples and key features in side-by-side heatmaps, each displaying data from one omics type. It consists of two main steps, detailed below.

#### Sample cluster detection

In multi-omics data, each omics type is a separate representation of the same samples, making it suitable for multi-view clustering (4). One main advantage of multi-view clustering is that it tends to reduce spurious correlations that are due to random noise or platform-specific technical artifacts, as it is highly unlikely that exact same erroneous effects are present across multiple datasets. OmicsAnalyst currently supports three multi-view clustering algorithms: spectral clustering (14), perturbation-based clustering (16) and similarity network fusion (13). The distinguishing features of these three methods are as follows. Spectral clustering makes use of eigenvalues derived from a similarity matrix to perform clustering based on fewer dimensions, which greatly increases the speed (17). OmicsAnalyst employs the *Spectrum* R package, which combines the advantages of spectral clustering with several other advanced techniques (14). Perturbation clustering assumes that reliable clusters are robust to small alterations to the data (4). OmicsAnalyst uses the perturbation clustering for data integration and disease subtyping (PINSplus) R package to support this approach (16). The similar network fusion (SNF) method involves fusing individual sample similarity matrices together using a rapid nearest neighbour approach (13). Since the associated *SNFtool* package does not support cluster detection, the spectral clustering is applied to the learned status matrix for this purpose.

#### Heatmap visual analytics

The results of clustering analysis can be intuitively explored via heatmaps, which use visual cues to show how samples are clustered and how feature abundances vary across samples. OmicsAnalyst implements an interactive joint-heatmap viewer where two different omics datasets can be visualized and analyzed simultaneously. The interactive visualization was implemented based on the INVEX heatmap viewer (18). It is organized into two main views consisting of an overview and a focus view for each omics data. The overview heatmap displays the overall abundance patterns for all features. Users can click-and-drag to select a region of interest to be displayed in the focus view for a more detailed inspection. The annotation bars along the top indicate the original group memberships as well as the cluster memberships based on the selected multi-view clustering algorithm. Similar to the correlation network analysis, users can perform enrichment analysis on the features displayed in the focus view for each omics type, when features are annotated during data processing.

### Dimension reduction analysis track

The objective of this track is to perform dimension reduction, and then visually explore corresponding scores, loadings and biplots in interactive 3D scatter plots to understand high-level trends and associated key features. It consists of two main steps, detailed below.

#### Multi-omics dimension reduction

Many standard multivariate dimension reduction techniques do not perform well on multi-omics datasets, which typically have many more features than observations (*p* >> *n*) and a multicollinear structure. Multivariate regression, the foundation of many multivariate dimension reduction techniques, performs poorly in these cases and so special care has been taken to develop more robust techniques for multi-omics data integration (19–21). OmicsAnalyst provides five different methods including multiple co-inertia analysis (MCIA), consensus PCA (CPCA), projection to latent structures (PLS), Procrustes analysis, and data integration analysis for biomarker discovery using latent components (DIABLO) (10,11,22,23). In general, these algorithms aim to identify sets of components that capture maximum variance within individual datasets and maximum association across datasets. They can be distinguished by individual optimization and constraint criteria used to identify component sets across the omics datasets. More detailed information and comparisons on these methods are provided in our FAQs under the ‘Dimension Reduction Analysis’ tab.

#### Visual analytics based on 3D scatter plots

OmicsAnalyst offers an interactive 3D scatter plot viewer that can display sample space (score plot), feature space (loading plot), as well as a ‘merged’ space (biplot) that overlays sample and feature spaces in the same plot to showcase contributions of key feature to the overall patterns. The 3D scatter plot viewer is divided into four different sections. The left panel contains a top section (‘Settings’) for controlling the overall visual environment of the scatter plots. The middle section (‘Overall Pattern’) allows users to change the grouping of nodes based on different meta-data or clustering analysis. It offers extensive options such as colors, shapes, and highlighting effects for group visualization. The bottom section displays information related to the current selections. The main scatter plot viewer in the center displays the current view - score plot, loading plot or biplot which allow users to specify features of interest to be shown as arrows on top of sample space. Users can also overlay different metadata groups as ellipsoids on top of the feature space. The right panel is divided into top (‘Comparison Test’) and bottom (‘Enrichment Analysis’) sections to allow users to perform targeted statistical and functional analysis on the current selected groups or clusters, respectively. Click a row of the result tables, the corresponding feature(s) will be displayed as arrows in the current score plot.

### Case study: multi-omics analysis of human pregnancy

To facilitate users to explore different features of OmicsAnalyst, three example multi-omics datasets have been provided including one from the Cancer Genome Atlas (TCGA, https://www.cancer.gov/tcga), one from the STATegra (24) and one from a recent multi-omics study on human pregnancy (25). Here, we provide a case study using the proteomics and metabolomics datasets from the pregnancy study.

**Figure 2.**
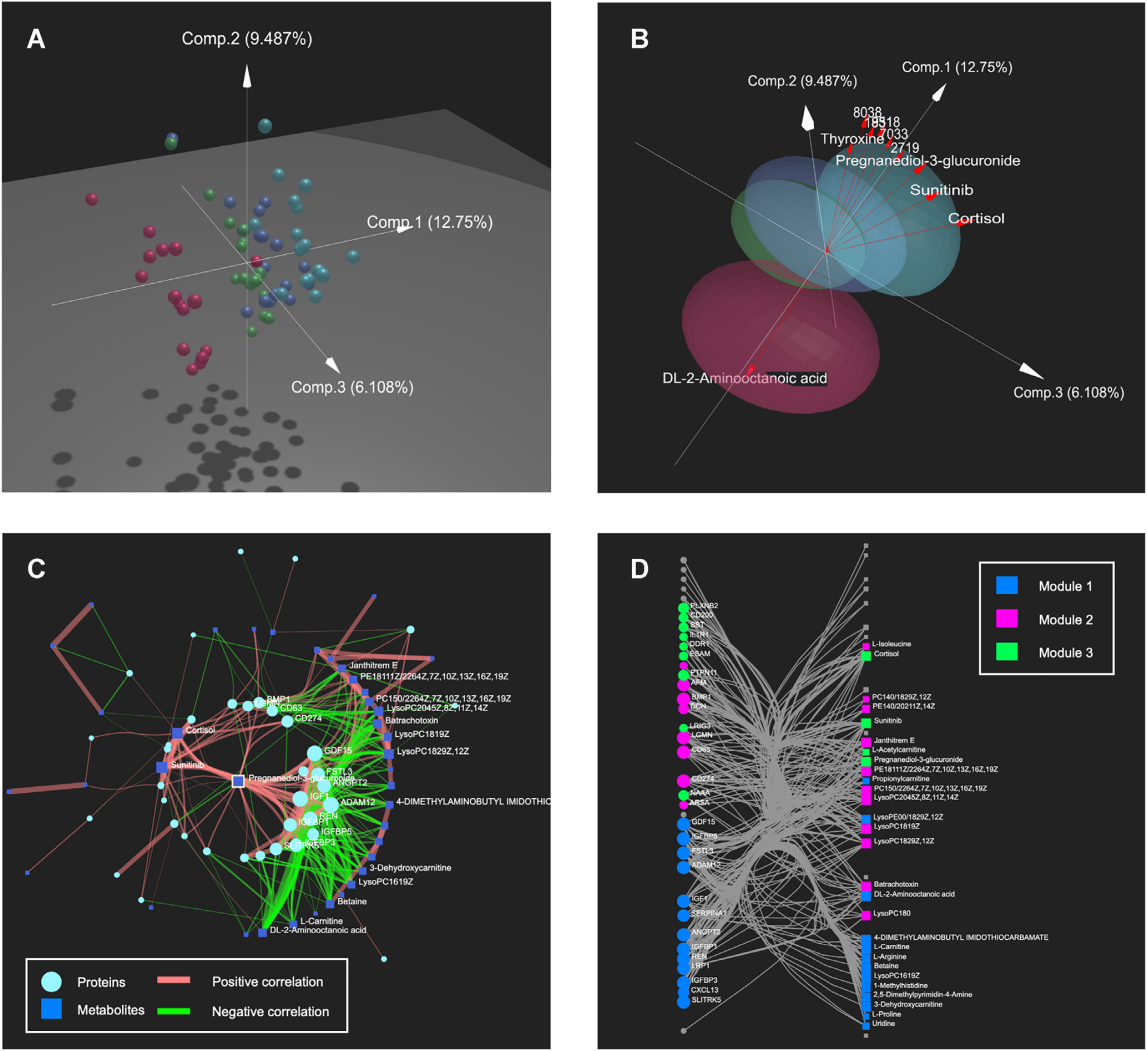
Example outputs from the case study. Dimension reduction was performed with DIABLO and results visualized with (**A**) 3D scatter plot of score plot, and (**B**) 3D biplot with elliptical summaries of sample groups (red = baseline, green = first trimester, dark blue = second trimester, light blue = third trimester) and the contributions of top five differentially expressed proteins and metabolites (red arrows). Correlation networks of features selected from the top three DIABLO components in (**C**) concentric circular layout, and (**D**) linear bipartite/tripartite layout, with modules detected by the ‘WalkTrap’ algorithm.

Various physiological systems are known to change predictably throughout pregnancy (26). This study was conducted to collect comprehensive molecular data (repeated samples from the first three trimesters and 6 weeks postpartum for baseline levels; *n* = 17 women) to build a predictive model for gestational age (25). Here, we re-analyze the proteomics and metabolomics data sets as a case study. Differential analysis was performed using ANOVA/t-tests with thresholds chosen to give ∼30% significant features (|log_2_FC| > 1; adjusted *P*-value < 0.005), and datasets were auto-scaled before integration. All three visual analytics tracks were used to gain complementary perspectives of the data. First, we used the ‘Free Exploration’ mode of the Heatmap Visual Analytics track to understand patterns present in individual omics. While the baseline samples form a weak cluster, samples from the three trimesters are very mixed. Next, we computed the multi-dimensional components that best separated the sample groups using DIABLO and explored the results with 3D scatter plots. The global structure confirms what we expect, with baseline samples well distinguished from those collected during pregnancy, and samples collected during later trimesters located further away from the baseline (Figure 2A). The biplot overlays the sample space with the top features that most contribute to the separation (Figure 2B), in this case highlighting several proteins and metabolites that are consistent with the biology of pregnancy. Three out of the five top metabolites are associated with hormones that are elevated during pregnancy (thyroxine, pregnanediol-3-glucuronide, and cortisol). One of the top proteins (ADAM12) is a serum marker for pregnancy, two (GDF15 and GPC3) are encoded by genes that have high expression in placenta relative to other tissues, and one is angiotensin (AGT), a hormone known to be elevated during pregnancy (27). All feature arrows point in the same direction, except for the dl-2-aminooctinoic acid metabolite. Finally, we used correlation networks to visualize relationships between key features from the top three DIABLO components. The network has a central cluster of proteins that are positively correlated with the proteins and metabolites on the left, and negatively correlated with the metabolites on the right (Figure 2C). Inspecting several individual features shows that the structure is consistent with Figure 2B: the central proteins and positively correlated metabolites contain many of the previously highlighted biplot features (ADAM12, Cortisol, Sunitinib, and Pregnanediol-3-glucuronide) while one of the negatively correlated metabolites is dl-2-aminooctinoic acid, the lone biplot feature that pointed in the opposite direction. Network module analysis with the ‘WalkTrap’ algorithm resulted in three modules, all of which contained both proteins and metabolites (Figure 2D). The blue module was statistically significant, and enrichment analysis revealed that it is significantly enriched for the Reactome pathway ‘Regulation of Insulin-like Growth Factor (IGF) transport and uptake by Insulin-like Growth Factor Binding Proteins (IGFBPs)’. IGF is known to be elevated during pregnancy (28). This case study has illustrated the improved insights and rich biological context when multi-omics data and visual analytics are used together. More details and figures from the case study are available from the ‘Tutorial’ page (under the ‘Case Study’ tab) of OmicsAnalyst.

### Implementation

OmicsAnalyst was implemented based on JavaServer Faces (JSF) using the PrimeFaces (v10.0) library (http://primefaces.org/) and R (version 4.0.2). The visual analytics methods have been developed based on several JavaScript libraries including sigma.js (http://sigmajs.org) for 2D network visualization, and three.js (https://threejs.org) for 3D network and scatter plot visualization. The system is hosted on a Google Cloud *n1-highmem-8* instance (64 GB RAM and eight virtual CPUs with 2.6 GHz each).

### Comparison with other web-based tools

Table 1 shows the comparisons between OmicsAnalyst and three other web-based tools dedicated for multi-omics integration and analysis, including 3Omics (29), MiBiOmics (30) and OmicsNet (7). The 3Omics supports analysis of transcriptomics, proteomics and metabolomics data from human. It includes modules for correlation analysis, co-expression profiling, phenotype mapping and functional enrichment analysis. MiBiOmics tackles multi-omics integration through correlation analysis using WGCNA-based approach and dimension reduction analysis using MCIA and Procrustes analysis. Finally, OmicsNet uses *a priori* interaction information to construct multi-omics networks for genes, proteins, metabolites, miRNA, and transcription factors. The resulting network is interactively visualized in 3D space. OmicsAnalyst distinguishes itself by bringing together multivariate, data-driven feature selection and integration with innovative visual analytics for unbiased exploration and interrogation of complex multi-omics datasets.

**Table 1.** Comparison of OmicsAnalyst with other web-based tools. Symbols used for feature evaluations with ‘√’ for present, ‘-’ for absent and ‘+’ for a more quantitative assessment (more ‘+’ indicating better support). The URLs for each tool are given below

	OmicsAnalyst	3Omics	MiBiOmics	OmicsNet
Input format	Matrix	List, matrix	Matrix	List
**Data processing**				
Annotation	+++	+++	-	+++
Filtering	+++	-	+	-
Normalization	+++	-	+	-
Scaling	+++	-	+	-
Differential expression	+++	-	-	-
**Integration methods**				
Univariate correlation	✓	✓	✓	-
Partial correlation	✓	-	-	-
Similarity network fusion	✓	-	-	-
Spectral clustering	✓	-	-	-
Perturbation-based clustering	✓	-	-	-
MCIA	✓	-	✓	-
CPCA	✓	-	-	-
Procrustes analysis	✓	-	✓	-
PLS	✓	-	-	-
DIABLO	✓	-	-	-
**Visual analytics**				
Scatter plot	+++	-	+	-
Heatmap	+++	++	++	-
Network	+++	-	++	+++
*Contextual enrichment analysis*				
Metabolite sets	++	++	-	++
Gene sets	++	++	-	++
miRNA sets	++	-	-	-

• OmicsAnalyst: https://www.omicsanalyst.ca/

• 3Omics: https://3omics.cmdm.tw/

• MiBiOmics: https://shiny-bird.univ-nantes.fr/app/Mibiomics

• OmicsNet: https://www.omicsnet.ca/

## CONCLUSIONS

The motivation for OmicsAnalyst was to create an intuitive, web-based platform for multi-omics integration that allows researchers to fuse statistical and visual streams of evidence together to make more informed judgements. In particular, we implemented three distinct visual analytics tracks - feature correlation analysis coupled with networks, sample clustering analysis coupled with heatmaps, and dimension reduction analysis coupled with 3D scatter plots. In doing so, OmicsAnalyst enables users to dissect large and complex multi-omics datasets by facilitating pattern recognition and cognitive reasoning through powerful yet intuitive visual analytics.

## References

[1] Hasin Y. , Seldin M., Lusis A. Multi-omics approaches to disease. Genome Biol. 2017; 18:83. 28476144 10.1186/s13059-017-1215-1 PMC5418815

[2] Integrative H.M.P.R.N.C. The integrative human microbiome project: dynamic analysis of microbiome-host omics profiles during periods of human health and disease. Cell Host Microbe. 2014; 16:276–289. 25211071 10.1016/j.chom.2014.08.014 PMC5109542

[3] Chong J. , Xia J. Computational approaches for integrative analysis of the metabolome and microbiome. Metabolites. 2017; 7:62. 10.3390/metabo7040062 PMC5746742 29156542

[4] Rappoport N. , Shamir R. Multi-omic and multi-view clustering algorithms: review and cancer benchmark. Nucleic Acids Res. 2018; 46:10546–10562. 30295871 10.1093/nar/gky889 PMC6237755

[5] Kamburov A. , Cavill R., Ebbels T.M., Herwig R., Keun H.C. Integrated pathway-level analysis of transcriptomics and metabolomics data with IMPaLA. Bioinformatics. 2011; 27:2917–2918. 21893519 10.1093/bioinformatics/btr499

[6] Hernandez-de-Diego R. , Tarazona S., Martinez-Mira C., Balzano-Nogueira L., Furio-Tari P., Pappas G.J. Jr, Conesa A. PaintOmics 3: a web resource for the pathway analysis and visualization of multi-omics data. Nucleic Acids Res. 2018; 46:W503–W509. 29800320 10.1093/nar/gky466 PMC6030972

[7] Zhou G. , Xia J. OmicsNet: a web-based tool for creation and visual analysis of biological networks in 3D space. Nucleic Acids Res. 2018; 46:W514–W522. 29878180 10.1093/nar/gky510 PMC6030925

[8] Huang S. , Chaudhary K., Garmire L.X. More is better: recent progress in multi-omics data integration methods. Front Genet. 2017; 8:84. 28670325 10.3389/fgene.2017.00084 PMC5472696

[9] Akavia U.D. , Litvin O., Kim J., Sanchez-Garcia F., Kotliar D., Causton H.C., Pochanard P., Mozes E., Garraway L.A., Pe’er D. An integrated approach to uncover drivers of cancer. Cell. 2010; 143:1005–1017. 21129771 10.1016/j.cell.2010.11.013 PMC3013278

[10] Rohart F. , Gautier B., Singh A., Lê Cao K.-A.J.P.c.b. mixOmics: an R package for ‘omics feature selection and multiple data integration. 2017; 13:e1005752. 10.1371/journal.pcbi.1005752 PMC5687754 29099853

[11] Meng C. , Kuster B., Culhane A.C., Gholami A.M.J.B.b. A multivariate approach to the integration of multi-omics datasets. 2014; 15:162. 10.1186/1471-2105-15-162 PMC4053266 24884486

[12] Argelaguet R. , Velten B., Arnol D., Dietrich S., Zenz T., Marioni J.C., Buettner F., Huber W., Stegle O. Multi-omics factor analysis - a framework for unsupervised integration of multi-omics data sets. Mol. Syst. Biol. 2018; 14:e8124. 29925568 10.15252/msb.20178124 PMC6010767

[13] Wang B. , Mezlini A.M., Demir F., Fiume M., Tu Z., Brudno M., Haibe-Kains B., Goldenberg A.J.N.m. Similarity network fusion for aggregating data types on a genomic scale. 2014; 11:333. 10.1038/nmeth.2810 24464287

[14] John C.R. , Watson D., Barnes M.R., Pitzalis C., Lewis M.J. Spectrum: fast density-aware spectral clustering for single and multi-omic data. Bioinformatics. 2020; 36:1159–1166. 31501851 10.1093/bioinformatics/btz704 PMC7703791

[15] Kayano M. , Imoto S., Yamaguchi R., Miyano S. Multi-omics approach for estimating metabolic networks using low-order partial correlations. J. Comput. Biol. 2013; 20:571–582. 23899012 10.1089/cmb.2013.0043

[16] Nguyen H. , Shrestha S., Draghici S., Nguyen T.J.B. PINSPlus: a tool for tumor subtype discovery in integrated genomic data. 2019; 35:2843–2846. 10.1093/bioinformatics/bty1049 30590381

[17] Ng A.Y. , Jordan M.I., Weiss Y.J.A.i.n.i.p.s. On spectral clustering: analysis and an algorithm. 2002; 2:849–856.

[18] Xia J. , Lyle N.H., Mayer M.L., Pena O.M., Hancock R.E.W. INVEX—a web-based tool for integrative visualization of expression data. Bioinformatics. 2013; 29:3232–3234. 24078684 10.1093/bioinformatics/btt562 PMC3842763

[19] Cantini L. , Zakeri P., Hernandez C., Naldi A., Thieffry D., Remy E., Baudot A.J.N.c. Benchmarking joint multi-omics dimensionality reduction approaches for the study of cancer. Nat. Commun. 2021; 12:124. 33402734 10.1038/s41467-020-20430-7 PMC7785750

[20] Chauvel C. , Novoloaca A., Veyre P., Reynier F., Becker J.J.B.i.b. Evaluation of integrative clustering methods for the analysis of multi-omics data. 2020; 21:541–552. 10.1093/bib/bbz015 31220206

[21] Stein-O’Brien G.L. , Arora R., Culhane A.C., Favorov A.V., Garmire L.X., Greene C.S., Goff L.A., Li Y., Ngom A., Ochs M.F. et al. Enter the matrix: factorization uncovers knowledge from omics. Trends Genet. 2018; 34:790–805. 30143323 10.1016/j.tig.2018.07.003 PMC6309559

[22] Dixon P. VEGAN, a package of R functions for community ecology. J. Vegetat. Sci. 2003; 14:927–930.

[23] Meng C. , Basunia A., Peters B., Gholami A.M., Kuster B., Culhane A.C. MOGSA: integrative single sample gene-set analysis of multiple omics data. Mol. Cell Proteomics. 2019; 18:S153–S168. 31243065 10.1074/mcp.TIR118.001251 PMC6692785

[24] Gomez-Cabrero D. , Tarazona S., Ferreiros-Vidal I., Ramirez R.N., Company C., Schmidt A., Reijmers T., Paul V.V.S., Marabita F., Rodriguez-Ubreva J. et al. STATegra, a comprehensive multi-omics dataset of B-cell differentiation in mouse. Sci Data. 2019; 6:256. 31672995 10.1038/s41597-019-0202-7 PMC6823427

[25] Ghaemi M.S. , DiGiulio D.B., Contrepois K., Callahan B., Ngo T.T., Lee-McMullen B., Lehallier B., Robaczewska A., Mcilwain D., Rosenberg-Hasson Y. Multiomics modeling of the immunome, transcriptome, microbiome, proteome and metabolome adaptations during human pregnancy. Bioinformatics. 2019; 35:95–103. 30561547 10.1093/bioinformatics/bty537 PMC6298056

[26] Aghaeepour N. , Ganio E.A., Mcilwain D., Tsai A.S., Tingle M., Van Gassen S., Gaudilliere D.K., Baca Q., McNeil L., Okada R. An immune clock of human pregnancy. Sci. Immunol. 2017; 2:eaan2946. 28864494 10.1126/sciimmunol.aan2946 PMC5701281

[27] Irani R.A. , Xia Y. Seminars in nephrology. 2011; 31:Elsevier 47–58. 21266264 10.1016/j.semnephrol.2010.10.005 PMC3275085

[28] Yang M.-J. , Tseng J.-Y., Chen C.-Y., Yeh C.-C. Changes in maternal serum insulin-like growth factor-I during pregnancy and its relationship to maternal anthropometry. J. Chin. Med. Assoc. 2013; 76:635–639. 23948255 10.1016/j.jcma.2013.07.004

[29] Kuo T.-C. , Tian T.-F., Tseng Y.J. 3Omics: a web-based systems biology tool for analysis, integration and visualization of human transcriptomic, proteomic and metabolomic data. BMC Syst. Biol. 2013; 7:64. 23875761 10.1186/1752-0509-7-64 PMC3723580

[30] Zoppi J. , Guillaume J.-F., Neunlist M., Chaffron S. MiBiOmics: an interactive web application for multi-omics data exploration and integration. BMC Bioinformatics. 2021; 22:6. 33407076 10.1186/s12859-020-03921-8 PMC7789220

